# Susceptibility of Austrian Clinical *Klebsiella* and *Enterobacter* Isolates Linked to Patient-Related Data

**DOI:** 10.3389/fmicb.2016.00034

**Published:** 2016-02-05

**Authors:** Alexandra Badura, Gudrun Pregartner, Judith C. Holzer, Gebhard Feierl, Andrea J. Grisold

**Affiliations:** ^1^Institute of Hygiene, Microbiology and Environmental Medicine, Medical University of GrazGraz, Austria; ^2^Institute for Medical Informatics, Statistics and Documentation, Medical University of GrazGraz, Austria

**Keywords:** antibiotic resistance, *Klebsiella*, *Enterobacter*, Austria, statistical analysis

## Abstract

The aim of the study was to analyze the antimicrobial susceptibility of Austrian clinical *Klebsiella* sp. and *Enterobacter* sp. isolates linked to patient-related data over a time period from 1998 to 2014. The main findings of this study were (i) a marked difference of antibiotic susceptibility rates between different infection sites for both *Klebsiella* sp. and *Enterobacter* sp., (ii) significantly greater percentages of resistant isolates among both *Klebsiella* sp. and *Enterobacter* sp. in male patients compared to female patients and (iii) significantly greater percentages of resistant isolates among both *Klebsiella* sp. and *Enterobacter* sp. from hospital-derived samples compared to samples from the community. In conclusion, our statistical data analysis clearly indicated a strong association of patient-related data and *Klebsiella* sp. and *Enterobacter* sp. susceptibility profiles.

## Introduction

Whereas the antibiotic resistance crisis in Gram-positive pathogens largely seems to be kept under control, the emergence and dissemination of antibiotic resistance in Gram-negative pathogens risk getting out of control (Arya et al., 2008; Slama, 2008; Bush et al., 2011; Livermore, 2012). To gain useful information on overall trends in a specific region and alert for more effective prevention and control measures, surveillance studies based on large data sets are of particular importance in this field (World Health Organization [WHO], 2014). Multivariate statistical analysis further allows studying possible relationships between antimicrobial resistance data and patient-related factors; however, there are only a limited number of studies up to now on that issue mainly dealing with *Escherichia coli*. The main findings of these studies are that susceptibilities to several antimicrobial agents are strongly depending on infection site as well as patient location (hospital/community), gender and age (Livermore et al., 2003; Miguel Sahuquillo-Arce et al., 2011; Badura et al., 2015). Under the hypothesis that the same is true for *Klebsiella* sp. and *Enterobacter* sp., the aim of this study is to analyze a large amount of antibiotic resistance data (almost 39.000 isolates) from our geographical region according to patient-related factors. The application of multivariate statistical analysis enables us to detect significant associations between patient’s characteristics and the percentages of resistant isolates what is of key importance to further identify possible risk factors related to antibiotic-resistant *Klebsiella* and *Enterobacter* infections.

## Materials and Methods

A total of 24.024 *Klebsiella* sp. and 14.969 *Enterobacter* sp. isolates were included in the present analysis. The study design of this retrospective observational study (data origin, laboratory methods, and statistical analysis) is largely similar to our previous report on *E. coli* (Badura et al., 2015). Data were retrieved from the Laboratory of Medical Bacteriology and Mycology of the Medical University of Graz, Austria from January 1998 to March 2014. In the event of multiple isolates from one person in a year, only the first one was considered. For each isolate, patient–related data (age, gender), patient location (community/hospital) and culture site were obtained. Culture sites were subdivided into the following categories: urinary tract (UT), genital tract (GT), wounds (WOU), respiratory tract (RES), and blood (BLO); patient age was categorized as follows: <1, 1–9, 10–19, 20–29, 30–39, 40–49, 50–59, 60–69, 70–79, > = 80 years.

All isolates had been tested for antibiotic susceptibilities in the routine microbiology laboratory in the same way as reported in a previous study using the disk diffusion method or a VITEK 2 system (bioMérieux, Marcy l’Etoile France) (Badura et al., 2015). From January 1st, 1998 to May 31st, 2011 results were interpreted using the criteria recommended by the Clinical and Laboratory Standards Institute (CLSI), formerly known as National Committee for Clinical Laboratory Standards (NCCLS) (CLSI, 2012). From June 1st, 2011 results were interpreted using the criteria recommended by the European Committee on Antimicrobial Susceptibility Testing (EUCAST breakpoint tables v1.2, 2011) in its respective current version. For this study, resistance to the following 12 antibiotic agents was analyzed: amikacin (AN), amoxicillin-clavulanic acid (AMC; analyzed for *Klebsiella* sp. only), ceftazidime (CAZ), ciprofloxacin (CIP), cefotaxime (CTX), nitrofurantoin (NF; analyzed for urinary tract isolates only), gentamicin (GM), imipenem (IPM), mecillinam (MEC; analyzed for *Klebsiella* sp. urinary tract isolates only); meropenem (MEM), piperacillin/tazobactam (PT), and trimethoprim/sulfamethoxazole (SXT). Resistant and intermediate resistant isolates were combined.

### Statistical Analysis

For each of the two microbes and for each antibiotic under consideration, the absolute and relative frequencies of resistant samples were determined in total, per culture site, patient location, age group, gender and year of acquisition. In order to assess the influence of the covariates on antibiotic resistance, multivariable logistic regression analysis was performed. For binary covariates, odds ratios and 95% confidence intervals are presented and are adjusted for year of acquisition, patient gender, patient age, patient location and resistance phenotype; for culture site a model containing the aforementioned covariates as well as cultures site has been tested against a model without culture site and p values from the respective Chi-square test are presented. Statistical analysis was performed using the R package, version 3.1.1 and SPSS, version 21.0 (R Development Core Team, 2008; IBM Corp, 2012).

### Ethics Statement

The study was approved by the Ethics Committee of the Medical University of Graz (26-502 ex 13/14); patient records were anonymized prior to analysis.

## Results

The underlying data basis for the present analysis is shown in **Table 1.** In total, 38.993 clinical isolates of *Klebsiella* sp. and *Enterobacter* sp. were included in the statistical analysis.

**Table 1:** Data basis for the statistical analysis.

	Patient gender (%)		Patient location (%)		Number of isolates
			
	Female	Male	Community	Hospital	
*Klebsiella* sp.	59.4	40.5	52.3	47.7	24024
*Enterobacter* sp.	52.8	47.1	44.0	56.0	14969
Total					38993


### Species Distribution

Within the *Klebsiella* samples, *K. pneumoniae* (61%) and *K. oxytoca* (27.1%) were the most frequently recovered species; within the *Enterobacter* samples, *E. cloacae* complex (59.2%) and *E. aerogenes* (15.4%) were the most frequently recovered species.

### Overall and Annual Resistance Percentages

**FIGURE 1:**
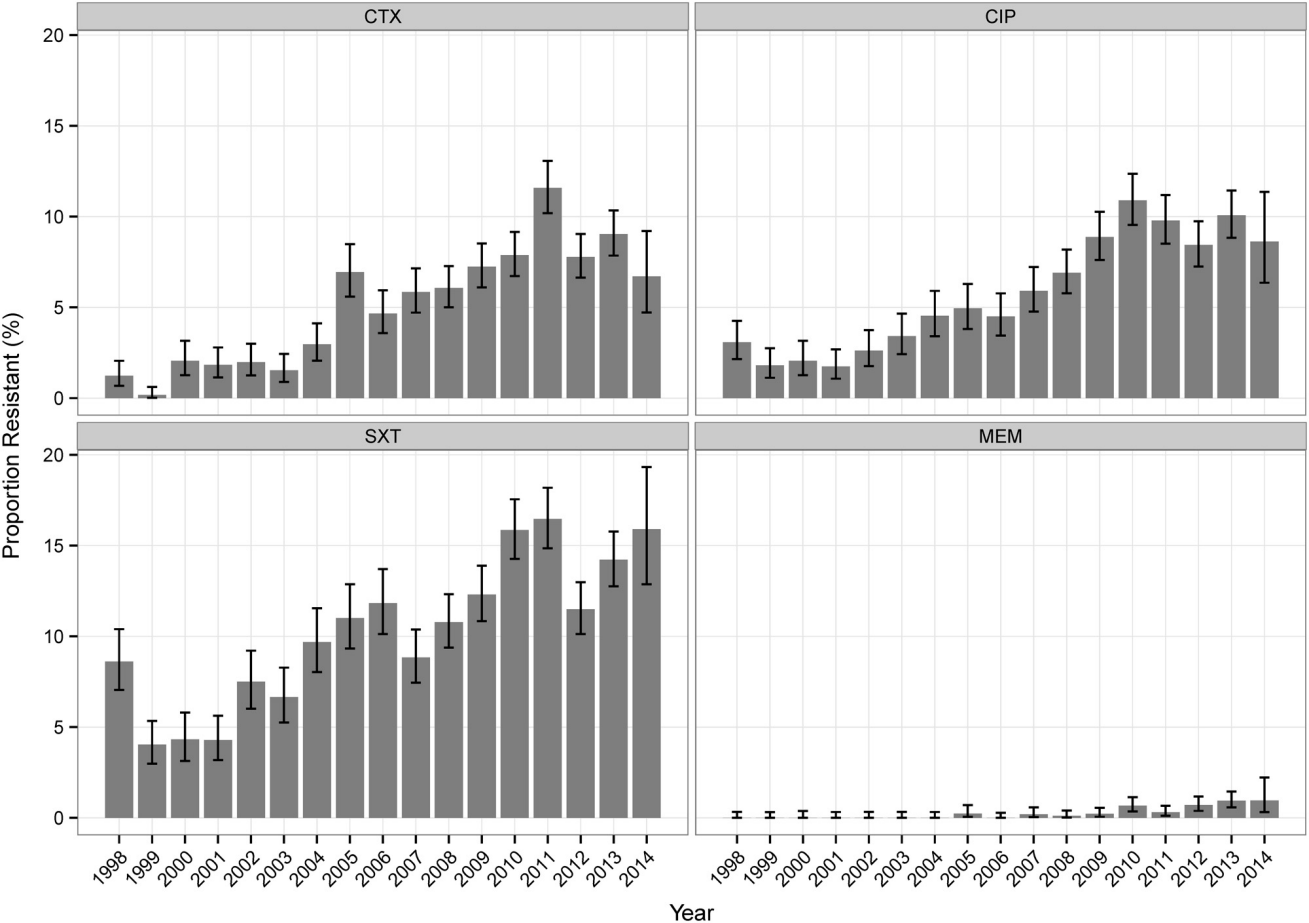
**Annual antibiotic resistance percentages of *Klebsiella* sp. for selected antibiotics**.

**Table 2** shows the overall resistance percentages of all tested isolates. For *Klebsiella* sp. isolates, NF (analyzed for UT isolates only) and SXT showed the highest values, for *Enterobacter* sp. the highest values were found for NF, CAZ, PT, and CTX. For *Klebsiella* sp. isolates, most antibiotics (CAZ, CTX, NF, CIP, SXT, IPM, and MEM) showed increasing percentages of resistant isolates over time (**Figure 1**, **Supplemental Table S1**). The percentages of resistant *Enterobacter* sp. isolates did not change considerably over the investigational period for the majority of antibiotic agents (**Supplemental Table S1**).

**Table 2:** Overall resistance percentages of *Klebsiella* sp. and *Enterobacter* sp.

Antibiotic	*Klebsiella* sp.		*Enterobacter* sp.	
		
	%R	n/total	%R	n/total
AN	3.47	366/10550	0.96	78/8094
AMC	9.44	2264/23979		
CAZ	7.19	1230/17102	20.55	2341/11389
CIP	6.43	1543/23995	5.2	778/14961
CTX	5.76	1370/23765	19.87	2943/14811
NF	24.43	2297/9404	30.71	1502/4891
GM	6.66	1458/21889	2.76	392/14216
IPM	0.89	60/6747	0.54	26/4839
MEC	3.73	120/3220		
MEM	0.67	70/10511	0.28	22/7720
PT	6.98	1270/18186	20.17	2505/12418
SXT	10.82	2595/23990	6.22	930/14958


*AN, amikacin; AMC, amoxicillin-clavulanic acid; CAZ, ceftazidime; CIP, ciprofloxacin; CTX, cefotaxime; NF, nitrofurantoin; GM, gentamicin; IPM, imipenem; MEC, mecillinam; MEM, meropenem; PT, piperacillin/tazobactam; SXT, trimethoprim/sulfamethoxazole; %R, percentages of non-susceptible isolates.*

### Resistance Percentages According to Patient-Related Data

Results of the multivariate analysis of the antibiotic resistance patterns according to culture sites for both pathogens are presented in **Table 3.** The findings reveal the lowest resistance percentages from samples originating from the male or female genital tract (GT). For *Klebsiella* sp. isolates, the highest resistance percentages for almost all antibiotics analyzed occurred in blood infections. For *Enterobacter*, the highest resistance percentages were found in isolates originating from blood (CAZ, CTX, GM, PT, SXT), and urinary tract infections (AN, CIP) (**Table 3**).

**Table 3:** Resistance percentages of *Klebsiella* sp. and *Enterobacter* sp. according to culture sites.

	*Klebsiella* sp. %R (n/total)						*Enterobacter* sp. %R (n/total)					
		
	UT	GT	WOU	RES	BLO	*p*-value^a^	UT	GT	WOU	RES	BLO	*p*-value^a^
AN	4.2 (32/766)	0.1 (1/1648)	1.4 (37/2657)	1.7 (48/2867)	0 (0/103)	0.022	5.7 (22/388)	0 (0/767)	0.5 (13/2629)	0.4 (10/2467)	0 (0/68)	<0.001
AMC	6.9 (845/12264)	2.7 (63/2363)	9.7 (298/3086)	10.7 (357/3347)	14.3 (17/119)	<0.001						
CAZ	7.9 (486/6156)	0.8 (16/1893)	5.2 (152/2923)	5.9 (190/3228)	10.1 (12/119)	<0.001	21.8 (611/2806)	6.4 (56/871)	17.9 (513/2869)	19.9 (553/2778)	36 (31/86)	<0.001
CIP	7.6 (937/12262)	1.6 (39/2365)	6.9 (213/3088)	5.9 (198/3354)	10.1 (12/119)	<0.001	7.4 (436/5887)	0.7 (7/1075)	5 (153/3039)	4 (115/2874)	4.7 (4/86)	<0.001
CTX	4.3 (518/12044)	1 (24/2364)	5.2 (161/3084)	5.6 (189/3350)	10.9 (13/119)	0.011	19.3 (1111/5747)	6.8 (73/1076)	18.2 (553/3038)	19.7 (564/2865)	36 (31/86)	<0.001
GM	3.5 (351/10162)	0.7 (17/2363)	3.9 (119/3087)	5.9 (198/3352)	4.2 (5/119)	<0.001	1.7 (86/5144)	0.1 (1/1076)	1.2 (37/3039)	1.3 (36/2872)	2.3 (2/86)	<0.001
IPM	0.2 (2/1110)	0 (0/814)	0.8 (12/1495)	1.1 (20/1755)	3.7 (3/81)	0.013	0.2 (1/583)	0 (0/306)	0.5 (7/1380)	0.3 (5/1476)	0 (0/54)	0.025
MEM	0.2 (5/2101)	0 (0/1124)	0.7 (15/2286)	1 (24/2414)	3.7 (4/108)	<0.001	0.3 (3/931)	0 (0/580)	0.2 (4/2344)	0.1 (3/2076)	0 (0/80)	0.003
PT	5.9 (421/7102)	1.4 (30/2121)	7.8 (230/2948)	9.1 (288/3156)	12.7 (15/118)	<0.001	16.3 (805/4934)	22.7 (204/899)	22.6 (539/2390)	23.4 (567/2426)	38 (27/71)	<0.001
SXT	12.6 (1551/12263)	4.2 (100/2365)	8 (248/3085)	7.2 (242/3354)	9.2 (11/119)	<0.001	6 (354/5881)	6.2 (67/1076)	6 (182/3041)	6.3 (182/2873)	14 (12/86)	0.221


*UT, urinary tract; GT, genital tract; WOU, wounds; RES, respiratory tract; BLO, blood; AN, amikacin; AMC, amoxicillin-clavulanic acid; CAZ, ceftazidime; CIP, ciprofloxacin; CTX, cefotaxime; NF, nitrofurantoin; GM, gentamicin; IPM, imipenem; MEM, meropenem; PT, piperacillin/tazobactam; SXT, trimethoprim/sulfamethoxazole. ^a^The p-values stem from a goodness-of-fit test comparing a model including all variables against a reduced model including all variables except for culture site.*

Antibiotic resistance patterns in relation to patient gender are shown in **Table 4.** Resistance percentages for almost all antibiotic agents analyzed are higher in male patients. A significant difference can be shown for all antibiotics except for AN for *Klebsiella* sp. isolates and for AN, GM, IPM, MEM, and PT for *Enterobacter* sp., respectively. Antibiotic resistance patterns in relation to patient location are shown in **Table 5.** Resistance percentages for almost all antibiotic agents analyzed are significantly higher in hospital treated patients. This is true for both *Klebsiella* sp. and *Enterobacter* sp. isolates.

**Table 4:** Antibiotic resistance percentages of *Klebsiella* sp. and *Enterobacter* sp. according to patient gender.

	*Klebsiella* sp. %R (n/total)			*Enterobacter* sp. %R (n/total)		
		
Antibiotic	Female	Male	OR^a^	Female	Male	OR^a^
AN	3.2 (174/5396)	3.7 (192/5141)	1.16 [0.95, 1.44]	0.8 (28/3731)	1.1 (50/4355)	1.43 [0.89, 2.34]
AMC	7.4 (1053/14245)	12.5 (1210/9715)	1.78 [1.63, 1.94]			
CAZ	5.9 (552/9398)	8.8 (677/7688)	1.55 [1.38, 1.74]	17.7 (990/5598)	23.3 (1350/5782)	1.13 [1.03, 1.25]
CIP	4.5 (647/14248)	9.2 (895/9728)	2.13 [1.92, 2.37]	3.3 (263/7899)	7.3 (515/7050)	2.2 [1.88, 2.57]
CTX	4.3 (611/14093)	7.9 (758/9653)	1.88 [1.68, 2.1]	17.4 (1361/7812)	22.6 (1580/6987)	1.1 [1.01, 1.2]
NF	22.4 (1392/6208)	28.3 (905/3196)	1.37 [1.24, 1.51]	28.2 (837/2972)	34.7 (665/1919)	1.23 [1.09, 1.4]
GM	4.8 (613/12791)	9.3 (844/9079)	2.04 [1.83, 2.27]	2.3 (170/7416)	3.3 (222/6788)	1.11 [0.9, 1.38]
IPM	0.5 (17/3464)	1.3 (43/3273)	2.7 [1.57, 4.87]	0.5 (12/2255)	0.5 (14/2578)	0.8 [0.37, 1.76]
MEC	3 (60/2012)	5 (60/1208)	1.62 [1.11, 2.36]			
MEM	0.4 (19/5174)	1 (51/5326)	2.07 [1.24, 3.61]	0.3 (9/3509)	0.3 (13/4206)	1 [0.43, 2.43]
PT	5.3 (545/10257)	9.2 (724/7912)	1.35 [1.19, 1.52]	19.7 (1286/6523)	20.7 (1216/5887)	1.01 [0.93, 1.11]
SXT	9.3 (1321/14245)	13.1 (1273/9726)	1.3 [1.19, 1.43]	5.8 (457/7897)	6.7 (472/7049)	1.16 [1.01, 1.33]


*AN, amikacin; AMC, amoxicillin-clavulanic acid; CAZ, ceftazidime; CIP, ciprofloxacin; CTX, cefotaxime; NF, nitrofurantoin; GM, gentamicin; IPM, imipenem; MEC, mecillinam; MEM, meropenem; PT, piperacillin/tazobactam; SXT, trimethoprim/sulfamethoxazole. OR, odd ratio*

**Table 5:** Antibiotic resistance percentages of *Klebsiella* sp. and *Enterobacter* sp. according to patient location.

	*Klebsiella* sp. %R (n/total)			*Enterobacter* sp. %R (n/total)		
		
Antibiotic	Outpatient	Hospital	OR^a^	Outpatient	Hospital	OR^a^
AN	0.3 (10/2894)	4.6 (356/7656)	4.18 [2.24, 8.71]	0.6 (11/1782)	1.1 (67/6312)	1.39 [0.74, 2.83]
AMC	4.8 (602/12557)	14.6 (1662/11422)	2.43 [2.18, 2.71]			
CAZ	4.5 (303/6703)	8.9 (927/10399)	1.32 [1.04, 1.69]	10.2 (369/3607)	25.3 (1972/7782)	2.97 [2.63, 3.37]
CIP	5.6 (700/12558)	7.4 (843/11437)	1.08 [0.96, 1.23]	4.5 (297/6587)	5.7 (481/8374)	1.22 [1.05, 1.43]
CTX	2.7 (335/12552)	9.2 (1035/11213)	2.39 [1.8, 3.18]	11.8 (775/6584)	26.4 (2168/8227)	2.67 [2.44, 2.93]
NF	21 (1376/6557)	32.3 (921/2847)	1.77 [1.59, 1.96]	26.1 (872/3339)	40.6 (630/1552)	1.82 [1.6, 2.07]
GM	2 (205/10465)	11 (1253/11424)	2.86 [2.4, 3.42]	0.7 (40/5847)	4.2 (352/8369)	4.48 [3.23, 6.39]
IPM	0.2 (4/1863)	1.1 (56/4884)	2.55 [1.01, 8.59]	0 (0/1032)	0.7 (26/3807)	^∗^
MEC	3.3 (78/2393)	5.1 (42/827)	1.3 [0.87, 1.93]			
MEM	0.2 (4/2508)	0.8 (66/8003)	5.14 [2.09, 17.09]	0.1 (1/1534)	0.3 (21/6186)	5.79 [1.18, 104.62]
PT	3.4 (266/7892)	9.8 (1004/10294)	2.71 [2.34, 3.14]	18.3 (956/5219)	21.5 (1549/7199)	1.2 [1.09, 1.32]
SXT	9.5 (1190/12556)	12.3 (1405/11434)	0.95 [0.86, 1.05]	5.9 (387/6582)	6.5 (543/8376)	1.05 [0.91, 1.21]


*AN, amikacin; AMC, amoxicillin-clavulanic acid; CAZ, ceftazidime; CIP, ciprofloxacin; CTX, cefotaxime; NF, nitrofurantoin; GM, gentamicin; IPM, imipenem; MEC, mecillinam; MEM, meropenem; PT, piperacillin/tazobactam; SXT, trimethoprim/sulfamethoxazole. ^∗^Not estimable because of 0 resistant isolates in outpatient group. OR, odd ratio*

**FIGURE 2:**
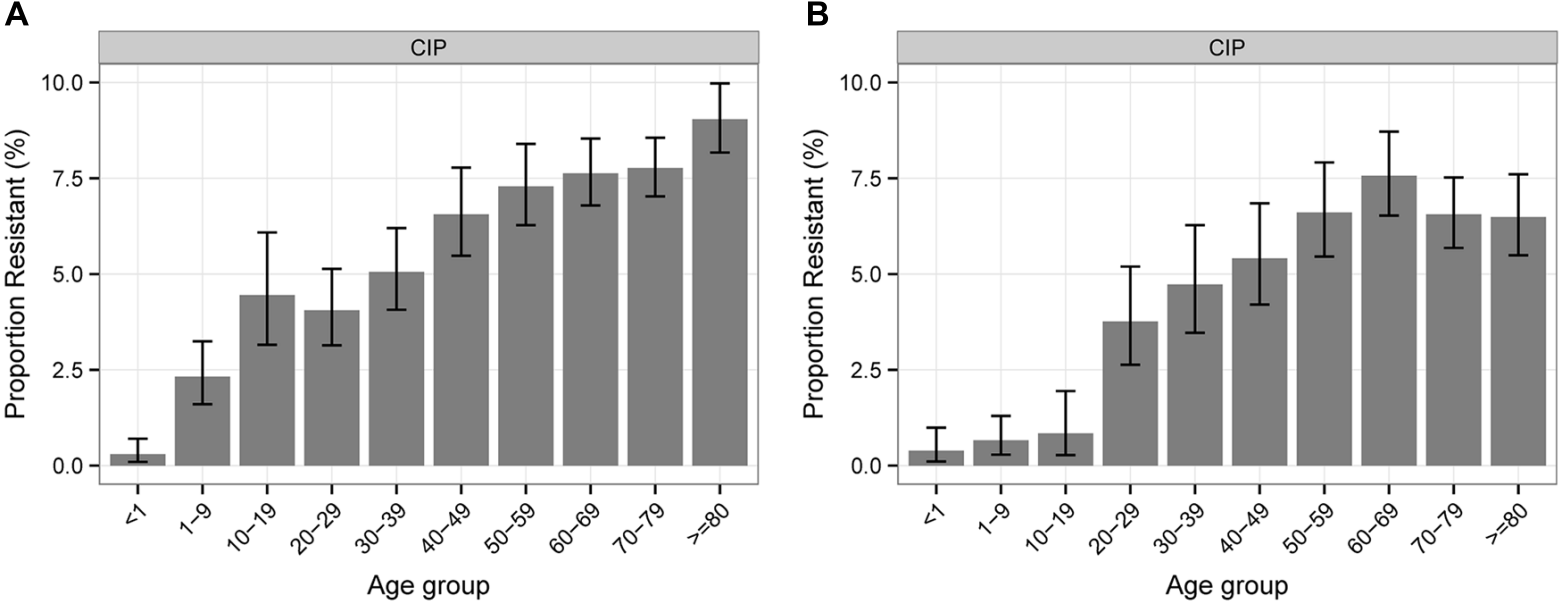
**Percentages of antibiotic resistance of *Klebsiella* sp. **(A)** and *Enterobacter* sp. **(B)** to CIP according to patient age**.

Regarding the association between antibiotic resistance percentages of *Klebsiella* sp. and *Enterobacter* sp. isolates and patient age, the majority of antibiotics show a balanced distribution except for CIP where a constant increase with age can be shown for both pathogen groups (**Figure 2**).

## Discussion

We here report an observational study whose aim is to analyze the susceptibility of Austrian clinical *Klebsiella* sp. and *Enterobacter* sp. isolates linked to patient-related data. The underlying database for this statistical analysis covers the period from 1998 to 2014. A major limitation of the present study is that it was not possible to link the data to severity of disease and patient outcome as these data were not available. Additionally, information about the patient’s length of the hospital stay was not included in the analysis which would have been useful for the classification as hospital or community acquired infections. In the present work, infections were categorized depending on the patient’s location at the moment the clinical sample was obtained. The passage from CLSI to EUCAST guidelines in 2011 in Austria may further bias data comparability. Several authors have reported an increase of antibiotic resistance rates for certain *Enterobacteriaceae* species/drug combinations following EUCAST breakpoints implementation (Hombach et al., 2012; Wolfensberger et al., 2013). However, we did not observe any artificial rise of antibiotic resistance at that time indicating that the change of interpretation criteria does not apparently impact the results of the large data set analyzed.

In general, surveillance is a substantial basis in the combat against antimicrobial resistance in both community and hospital settings; a large number of resistance surveillance reports thus exist about *Klebsiella* sp. and *Enterobacter* sp. isolates from various parts of the world describing the current resistance situation (Jones et al., 2004; Bedenic et al., 2010; Gales et al., 2012; Badal et al., 2013; Kaye and Pogue, 2015). Furthermore, studies dealing with the association of antimicrobial resistance data and specific factors depending on the host are of major importance with regard to the identification of possible risk factors related to antibiotic-resistant infections. Several studies aimed to find various patient demographics and clinical factors associated with the presence of antibiotic-resistant *Enterobacteriaceae* isolates, however, most of them involve particular infections, patient cohorts, anatomic sites or specific resistance problems. These studies thus mostly cover a small amount of isolates consequently limiting statistical data processing (O’Fallon et al., 2010; Metan et al., 2013; Ivady et al., 2015; Moini et al., 2015; Oliveira et al., 2015). Here we present a comprehensive statistical analysis on associations between patient-related data and the percentages of antibiotic resistant *Klebsiella* sp. and *Enterobacter* sp. isolates referring on a large database with almost 39.000 isolates from Austria. The main findings of this study are (i) a marked difference of antibiotic susceptibility rates between different infection sites for both *Klebsiella* sp. and *Enterobacter* sp., (ii) significantly greater percentages of resistant isolates among both *Klebsiella* sp. and *Enterobacter* sp. in male patients compared to female patients and (iii) significantly greater percentages of resistant isolates among both *Klebsiella* sp. and *Enterobacter* sp. isolates from hospital-derived samples compared to samples from the community.

To our knowledge, this is the first report to compare percentages of antibiotic resistant *Klebsiella* sp. and *Enterobacter* sp. isolates from different infection sites within a specific region. The highest resistance percentages for the majority of antibiotics analyzed can be shown for both pathogen groups originating from blood infections. This is in accordance with previous statistical analyses on the association of the infection site and *Escherichia coli* susceptibility profiles (Miguel Sahuquillo-Arce et al., 2011; Badura et al., 2015). As reported for *Escherichia coli*, *Klebsiella* sp. and *Enterobacter* sp. isolates originating from the GT generally show the lowest resistance percentages indicating that detection from this culture site often represents the normal patient flora. Interestingly, antibiotic-resistant *Klebsiella* sp. and *Enterobacter* sp. infections within our study collection derive mostly from men. The same association between patient sex and antibiotic resistance was previously shown for *Escherichia coli*, however, the underlying cause remains as yet largely unclear (Livermore et al., 2003; Miguel Sahuquillo-Arce et al., 2011; Badura et al., 2015). Similar to the results from several other studies dealing with antibiotic resistance percentages of *Enterobacteriaceae* in community and hospital settings we found significantly greater resistance rates for both pathogen groups amongst the hospital-derived isolates suggesting that the problem of antibiotic resistance amongst Gram-negative pathogens is still focused on hospital settings in our region (Boyd et al., 2008; Ferjani et al., 2015; McKay and Bamford, 2015).

## Conclusion

Our statistical data analysis clearly indicates a strong association between patient characteristics (gender, localization, infection site) and *Klebsiella* sp. and *Enterobacter* sp. susceptibility profiles. To gain further insight in the correlation of bacterial antibiotic resistance and its host, additional studies analyzing large databases of microbiological information are of crucial importance ultimately influencing local antimicrobial therapy guidelines.

## Author Contributions

AB: general conception, study design, data acquisition, data analysis, data interpretation, writing of the manuscript; GF: data interpretation, revising of the manuscript; JH and GP: statistical analysis, data interpretation, revising of the manuscript; AG: data interpretation, revising of the manuscript.

## Conflict of Interest Statement

The authors declare that the research was conducted in the absence of any commercial or financial relationships that could be construed as a potential conflict of interest.

## Supplementary Material

**TABLE S1 | Annual resistance percentages of *Klebsiella* sp. and *Enterobacter* sp.**
